# Thymic Microenvironment and Lymphoid Responses to Sublethal Irradiation

**DOI:** 10.1155/1995/14923

**Published:** 1995

**Authors:** Elise S. Randle-Barrett, Richard L. Boyd

**Affiliations:** Department of Pathology and Immunology Monash Medical School Commercial Road Prahran Victoria 3181 Australia

**Keywords:** T-cell, thymus, irradiation, thymocyte

## Abstract

Sublethal irradiation of the murine thymus has been a useful tool for depleting the thymus of dividing immature thymocyte subsets, to sequence thymocyte differentiation events occurring from radiation-resistant precursors. This massive reduction in thymocytes also represents a model in which the bidirectional interplay between the thymic stromal cells and lymphocytes can be investigated. The purpose of this study was thus twofold: to precisely map the initiation of thymopoiesis as a prelude to assessing the effects of injected mAb to novel thymic antigens; and to use a panel of mAbs to determine the alterations in the thymic stroma during the T-cell depletion and reconstitution phases. The striking finding from this study was that following T-cell depletion, there was a marked upregulation of specific stromal antigens, which retracted with the reappearance of T cells. Thus, following sublethal irradiation, there are modifications in the thymic microenvironment that may be necessary to support renewed thymopoiesis and the complete restoration of the thymus involved the synchronous development of both the stromal and lymphocytic components.


					Developmental Immunology, 1995, Vol 4, pp. 101-116
Reprints available directly from the publisher
Photocopying permitted by license only
(C) 1995 Harwood Academic Publishers GmbH
Printed in Singapore
Thymic Microenvironment and Lymphoid Responses to
Sublethal Irradiation
ELISE S. RANDLE-BARRETT"t and RICHARD L. BOYD
.Department of Pathology and Immunology, Monash Medical School, Commercial Road, Prahran, 3181, Vic, Australia
Sublethal irradiation of the murine thymus has been a useful tool for depleting the thymus
of dividing immature thymocyte subsets, to sequence thymocyte differentiation events
occurring from radiation-resistant precursors. This massive reduction in thymocytes also
represents a model in which the bidirectional interplay between the thymic stromal cells
and lymphocytes can be investigated. The purpose of this study was thus twofold: to
precisely map the initiation of thymopoiesis as a prelude to assessing the effects of injected
mAb to novel thymic antigens; and to use a panel of mAbs to determine the alterations in
the thymic stroma during the T-cell depletion and reconstitution phases. The striking
finding from this study was that following T-cell depletion, there was a marked upregu-
lation of specific stromal antigens, which retracted with the reappearance of T cells. Thus,
following sublethal irradiation, there are modifications in the thymic microenvironment that
may be necessary to support renewed thymopoiesis and the complete restoration of the
thymus involved the synchronous development of both the stromal and lymphocytic
components.
KEYWORDS: T-cell, thymus, irradiation, thymocyte.
INTRODUCTION
The thymic microenvironment consists of special-
ized cells that are both epithelial and nonepithelial
in nature. Together these cellular elements are or-
ganized into well-defined cortical and medullary
regions, throughout which developing thymocytes
reside (Boyd et al., 1993). As T cells mature within
the thymus, they migrate from the cortex to the
medulla and undergo phenotypic changes, includ-
ing the acquisition of TcR, cytokine receptors, and
the modulation of differentiation antigens such as
CD4 and CD8 (Boyd and Hugo, 1991). The contri-
bution of the thymic microenvironment toward this
complex process, and that which thymocytes them-
selves impart on the thymic microenvironment, is
gradually becoming defined. Thymic stromal cells
such as TNC, Mq, and DC form complexes with
developing thymocytes (Andrews and Boyd, 1985;
Kyewski, 1986; van Ewijk, 1988; Shortman and
Vremec, 1991; Gao et al., 1993) and stromal cell
lines instruct some, but not all, specific stages of
Corresponding author.
thymocyte development (Palacios et al., 1989; Nish-
imura et al., 1990; Tatsumi et al., 1990; Nagamine et
al., 1991; Hugo et al., 1992; Watanabe et al., 1992;
Anderson et al., 1993). Accordingly, damage to the
microenvironment itself via drug treatment or ion-
izing irradiation impairs thymocyte differentiation
(Adkins et al., 1988; Kanariou et al., 1989).
That the constitution and organization of the
thymic microenvironment are in turn dependent on
T-cell development has been well illustrated in a
number of experimental systems. In SCID mice and
in mice deficient in RAG-1 or p56lck, the medulla
fails to develop due to the absence of mature TcR
thymocytes (Shores et al., 1991; Molina et al., 1992;
Suhr et al., 1992; Ritter and Boyd, 1993; van Ewijk
et al., 1994). Similarly, in TcR knockout mice, where
thymocyte development is blocked beyond
CD4/CD8 cells, the medulla is greatly reduced in
size (Philpott et al., 1992; Palmer et al., 1993;
Mombaerts et al., manuscript in preparation). This
effect has also been demonstrated in mice that have
been injected with anti-CD3 mAb (Kyewski, 1991).
Furthermore, a thymocyte-stromal cell signaling
pathway in which medullary epithelial cells are
activated via the phosphorylation of a 90-kD
101
102 E.S. RANDLE-BARRETT and R.L. BOYD
medullary epithelial-cell glycoprotein upon contact
with CD4+CD8 thymocytes has been identified
(Couture et al., 1992). Thus, it is evident that a
bidirectional relationship exists between thymocytes
and stromal cells (reviewed by Ritter and Boyd,
1993).
In the present study, we have mapped thymocyte
reconstitution post-irradiation (PIrr) to ultimately
devize a model for examining the in vivo effects
specific mAbs have on thymopoiesis. Using a panel
of mAb-recognizing mouse thymic stromal (MTS)
antigens derived in this laboratory (Godfrey et al.,
1990; Tucek et al., 1992), we also observed the
effects of irradiation and the associated loss of T
cells on the thymic microenvironment. In agreement
with others (Huiskamp et al., 1983; Huiskamp and
van Ewijk, 1985; Adkins et al., 1988), the initial loss
of lymphocytes following irradiation resulted in the
collapse of the stromal architecture, in particular,
that of the cortex. Most importantly, however, it was
clearly illustrated that the thymic was "dynamic"
immediately following irradiation, with marked up-
regulation in the expression of specific cortical and
medullary stromal antigens. These selective changes
in the thymic stroma may have been as a direct
result of the irradiation itself, revealed as a conse-
quence of the rapid depletion of thymocytes or a
prerequisite for the initiation of thymopoiesis fol-
lowing irradiation.
RESULTS
Effect of Irradiation on Thymocyte
Subpopulations
Total Thymocyte Cell Number. Total cell yields dra-
matically decreased immediately following sublethal
irradiation (Fig. 1) from their normal adult value
(day 0) of 6.0+1.4x107 to the nadir of 4.2+
1.2 x 105, by day 5. Between days 6 and 7, however,
there was extensive thymocyte proliferation as in-
dicated by the tenfold increase in total cell number
to 4.4+0.31x 106, with an additional tenfold in-
crease in number by day 12 to 3.3 +0.2x 107.
CD3, CD4, and CD8 Expression. To assess thymocyte
subpopulations and their subsequent reconstitution,
thymocytes on days 1-14, 21, and 28 PIrr were
stained simultaneously for CD3, CD4, and CD8
antigen expression. By day 1, the thymus is virtually
devoid of CD4/CD8 double-positive (DP) thymo-
08
TOTAL CELL YIELDS POST IRRADIATION
07
06
05
2 4 6 1 lt2 14 1 ;8 20 2 24 26 28
DAYS POST IRRADIATION
FIGURE 1. Total thymocyte cell yields of normal adult CBA/
Call (day 0) mice and sublethally irradiated mice, 1-28 days
postirradiation. Results represent the mean + standard deviation
of four independent experiments, except for days 8, 9, and 13,
which are represented as the mean of two experiments.
cytes (Fig. 2), the small remnant population being
mainly of the CD4/CD8/CD3hi
subset (Fig. 3). The
resistance of mature populations to the irradiation
was further reflected in the mature (CD3/) CD4 and
CD8 single-positive populations. Although these
cells markedly increased in proportion within the
first 4 days (Figs. 2 and 3), they progressively
decreased in number (from 4.3+1.0x106 to
7.5+0.7x104 and 1.2+_0.4x106 to 1.8+_3.0x104,
respectively) until day 10, presumably due to emi-
gration (K. Kelly, personal communication). Simi-
larly, CD3+CD4-CD8 thymocytes were unaffected
proportionally (Fig. 3) but decreased in number
from 3.0_+0.8x105 to 1.0+1.0x104 by day 5.
The immature CD3-CD4-CD8- triple-negative
(TN) thymocytes were relatively increased initially,
but by day 3 had decreased in both proportion (Fig.
3) and cell number (from 9.0 + 3.0 x105 to
1.2 + 1.0 x 104), those remaining being the radiation-
resistant cells that subsequently give rise to thymic
reconstitution (Sharp and Thomas, 1977; Hiesch
and Revesz, 1979). Immature CD4 and CD8 single-
positive populations were also reduced tenfold in
number by day 4 (from 9.5+2.0x105 to
1.0+__0.5x104 and 4.0+2.0x105 to 0.3+0.1x10,
respectively) although unaffected in proportion
within the first 3 to 4 days (Fig. 3).
Thymic reconstitution began around day 4 with
sequential, progressive increases in CD3-CD4-
CD8- cells (Figs. 2 and 3), some of which progressed
to the immature single-positive and subsequently
THYMIC RECONSTITUTION AFTER IRRADIATION 103
ADULT (Day 0)
...ii::."
;.
!..":.'
.
(;1)8
9.0 87
1.5 2.5
TOTAL CD3
43 'ff l
DAY 1
76 2.0
2.0 20
"4.018.0 88
,h,.
DAY 4
70 3.0
7.0 20
5.0 15
180 7.0 18 175
DAY 5
15
120 165
,,,
DAY 6
40 29
13 18
DAY 7
7.0 88
2.5 2.5
DAY 12
4.0 94
0.4 1.6
.:.'::::,"
1..,"
CD8
DAY 28
9.0 84
4.03.0
.,:..::,-::.....'.:..
t60
133 17.0 49 48
13.0 52 40 8.0
FIGURE 2. Three-color FCM analysis of CD3, CD4, and CD8 antigen expression on thymocytes from nonirradiated adult CBA/CaH
mice and sublethally irradiated mice 1-28 days following irradiation. Definition of CD4/CD8 subsets and total CD3 expression are
based on cursor settings defined from nonirradiated adult CBA thymocytes. The results were obtained by analysis of 3.0 10 cells
and represent four to five independent experiments.
104 E.S. RANDLE-BARRETT and R.L. BOYD
CD4"CD8"CD3"
14
12
10 12 14 16 18 20 22 24 26 28 30
CD4+CD8+CD3"
6o]
" T
40
.L
10 12 14 16 18 20 22 24 26 28 30
CD4+CD8"CD3"
TT T
10 12 14 16 18 20 22 24 26 28 30
CD4+CD8+CD3
60.
40 T
20-
10 12 14 16 18 20 22 24 26 28 30
CD4"CD8+CD3
-[
2.4
10 12 14 16 18 20 22 24 26 28 30
CD4+CD8+CD3hi
6.
T
10 12 14 16 18 20 22 24 26 28 30
100
CD4+CD8"CD3+ CD4"CD8+CD3+
20-
15-
10-
5-
lt0-;2 ;4 ;618 202224 26 2 30
DAYS POST IRRADIATION
CD4-CD8+CD3
T
10 12 14 16 18 20 22 24 26 28 30
FIGURE 3. Mean percentage values +standard deviations for CD3/CD4/CD8-defined thymocyte populations 1-28 days following
sublethal irradiation. Day 0 represents adult CBA nonirradiated thymocytes. Results were obtained by FCM analysis of 3.0 x 104 cells
and represent three to five independent experiments.
CD4+CD8 cells. Thus, between days 5-7 following
irradiation, all immature thymocyte populations
markedly expanded. By day 7, the thymocyte profile
with respect to CD3, CD4, and CD8 expression
approximated that of the adult thymus (Fig. 2),
although it is not clear whether the mature single-
positive cells were remnant radiation-resistant cells
that had not yet migrated or were derived from de
novo production of differentiating precursors. From
day 7, all thymocyte populations increased in cell
number, and by day 21, normal adult values were
virtually attained (data not shown).
MTS 32, 33, 35 and 37. MTS 32, 33, 35, and 37
identify antigens with the unusual property of being
expressed on both thymocytes and thymic stromal
cells, thereby providing an additional phenotypic
profile of thymocytes distinct from that defined by
CD3, CD4, and CD8 expression (Godfrey et al.,
1990; Tucek et al., 1992). On thymocytes, MTS 32
stains all T cells, except a subset of CD3hiCD4
CD8- cells, which exhibit a Th0/Th2-type cytokine
profile (Vacari et al., 1994); MTS 33 and 37, which
detect ThB and HSA, respectively, stain immature
thymocytes and mature thymocytes, but are nega-
tive on peripheral T cells; MTS 35, detects the
antigen TSA-1/Sca-2, which is restricted to imma-
ture thymocytes and has an inverse expression to
CD3 (Godfrey et al., 1992). Figure 4 shows the effect
of sublethal irradiation on the four thymocyte
determinants. MTS 32 progressively decreased from
96% to 60% by day 4 PIrr, and then gradually
THYMIC RECONSTITUTION AFTER IRRADIATION 105
MTS32 MTS 33 (ThB)
100"
80
60
20
80
60
40
20
4
/
/
/
/
/
/
/
/
/
/
/
6
80"
60"
40"
20"
12 14 2
MTS 35 (TSA-1/Sca-2)
100'
80
/ 60
/
40
20
0
3 4 5 6 12 14
MTS 37 (HSA)
/I
A
DAYS POST IRRADIATION
FIGURE 4. Percentage of thymocyte subpopulations defined by MTS mAb for sham irradiated (filled-in bars) and sublethally
irradiated (lined bars) mice days 1-14 post-irradiation. Results represent the mean of two concordant experiments.
increased to approximately the adult values by day
12. In accordance with the dramatic loss of imma-
ture thymocytes, PIrr, ThB, TSA-1/Sca-2, and HSA
expression were markedly reduced from approxi-
mately 90% to 2-5% by day 4, despite the fact that
HSA and ThB are expressed on mature CD3 cells,
albeit at low levels, which are virtually the only cells
remaining after irradiation.
Effect of Irradiation on the Thymic
Microenvironment
The effects of sublethal irradiation on the thymic
microenvironment were examined using an exten-
sive panel of mAbs reactive with epithelial and
nonepithelial stromal cells and the vasculature, the
normal adult thymus reactivities have been de-
scribed elsewhere (Godfrey et al., 1990). The data
are summarized in Tables 1 to 4.
Epithelial Cells. Following irradiation, the loss of
rapidly dividing thymocytes and decrease in thymus
size were associated with a collapse of the thymic
stromal network, particularly that of the cortex. This
was well illustrated by anti-CD4 and anti-
cytokeratin labeling of the thymic epithelium (Fig.
5). Similarly, MTS 44 is pan-cortical epithelium, but
also weakly stains infrequent, isolated medullary
epithelial cells in the normal adult thymus. By 3
days PIrr, MTS 44 epithelial cells formed a compact
106 E.S. RANDLE-BARRETT and R.L. BOYD
TABLE 1.1
Epithelium
MTS Reference
mAb Thymus 12 h Day
POST-IRRADIATION
Day2 Day3 Day4 Day5 Day6 Day 12
44 C: Ep
M: Ep Sp
10 M & Sc: Ep
NC* NC* NC*
NC*
6 Pan Ep
(MHC-II) M: KNC & Thy Sp
C' Thy Sp
NC*
C: Outer Ep C: Outer Ep C: Outer Ep NC* NC
C: Ep C: Ep C: Ep C: Ep C: Ep NC* NC
M: Ep M: Ep M: Ep M: Ep M: Ep
C: confluent C: confluent C: confluent C: confluent C: confluent NC* NC
M: NC M: NC M: NC M: NC M: NC
9 C' Isol Ep & Thy Sp NC* C: confluent C: confluent C: confluent C: confluent C: patchy NC* NC
M: Ep & Thy Sp M: EP M: EP M: EP M: EP M: EP
Abbreviations: BV: blood vessels; C: cortex; ECM: extracellular matrix: Ep: epithelium; Isol: isolated; IVC: intravascular cells; KNC: keratin-negative cells; M: medulla; NC:
change, mAb reactivity reflects that of reference thymus; NC*: change, mAb reactivity reflects that of the reference thymus but stromal architecture is collapsed; PVS:
perivascular space; Sc: subcapsule; Sp: subpopulation; Thy: thymocytes.
TABLE 1.2
Vascular/Connective Tissue Associated Elements
MTS Reference
POST-IRRADIATION
mAb Thymus 12 h Day Day 2 Day 3 Day 4 Day 5 Day 6 Day 12
12 Capillaries NC* NC
15 Granular staining NC* NC
around BV, in PVS & Sc space
16 Basement membrane NC* NC
associated ECM
Abbreviations: See footnote for Table 1.
TABLE 1.3
Isolated Stromal Elements
POST-IRRADIATION
MTS Reference
mAb Thymus 12 h Day Day 2 Day 3 Day 4 Day 5 Day 6 Day 12
17 h M & Sc: Isol KNC NC C: KNC C: KNC M: Ep(HC); M: Ep(HC); M: Ep(HC); NC* NC
KNC KNC KNC
29 M: Isol Ep cells NC NC* NC* M: KNC M: KNC M: KNC NC* NC
Abbreviations: See footnote for Table 1.
TABLE 1.4
Thymocyte-Stromal-Cell Shared Antigens
MTS Reference
POST-IRRADIATION
mAb Thymus 12 h Day Day 2 Day 3 Day 4 Day 5 Day 6 Day 12
32 C: Thy & Ep NC* C: Isol Ep; C: Isol Ep; C: Isol Ep; C: Isol Ep; C: Isol Ep; C: Isol Ep; NC
Thy Thy Thy Thy Thy
33
(ThB)
35
(TSA-1/
Sca-2)
37
(HSA)
C: Thy NC* C: Isol Ep C: Isol Ep C: Isol Ep C: Isol Ep C: Isol Ep NC
M: Ep clusters M: Ep M: Ep M: Ep M: Ep M: Ep M: Ep
C: Thy NC* C: Isol Ep C: Isol Ep M: KNC; M: KNC; M: KNC; NC*
M: Isol Ep cells Isol Ep Isol Ep Isol Ep
C, M, & PVS: Isol NC* C: KNC C: KNC M: KNC M: KNC M: KNC
Ep & KNC, IVC C: Ep C: Ep C" Ep
& Thy
NC*
NC
NC
Abbreviations: See footnote for Table 1.
THYMIC RECONSTITUTION AFTER IRRADIATION 113
Thymocyte-Stromal Shared Antigens. By immunohis-
tology, MTS 32 normally detects a marker expressed
on cortical thymocytes and isolated epithelial cells;
the medulla by contrast is negative. By days 1-2
PIrr, the expressions of MTS 32 had vanished,
leaving weak isolated epithelial cell staining in the
cortex. Pockets of granular staining (possibly dead/
dying thymocytes) were also observed within epi-
thelial cell-free regions. From days 3-6, MTS 32
remained weakly localized to infrequent epithelial
cells and thymocytes within the cortex; reactivity
had normalized by day 12. MTS 33 (ThB), normally
reactive with cortical thymocytes and medullary
epithelial clusters, displayed no thymocyte reactiv-
ity, but the latter were more obvious from days 1-6
PIrr, presumably as a result of the absence of T cells.
Additionally, isolated clusters of weak ThB epithe-
lial cells were revealed in the subcapsular cortex
(Fig. 10).
In accordance with flow cytometry, MTS 35 (TSA-
1/Sca-2) and MTS 37 (HSA) were all negative on
thymocytes from days 1-5, their reactivity on both
cortical and medullary thymic stromal-cell subsets
being more exposed. By day 6, their staining pat-
terns had normalized.
DISCUSSION
In view of the recent realization that a major feature
of thymic organogenisis is the bidirectional, symbi-
otic development relationship between thymic lym-
phocytes and stromal cells, the purpose of this study
was to examine the responsiveness of these stromal
cells to a dramatic loss in T cells. That is, has the
microenvironment encompassed within the stromal
elements the flexibility to alter in response to the
need to reinitiate or enhance T-cell differentiation?
If so, can such a study reveal which specific stromal
molecules are involved in this important process?
In addition to the anticipated dramatic loss of
thymocytes, the most striking feature of the present
study was the enhanced staining of selective thymic
stromal-cell antigens following sublethal irradiation.
There are three plausible explanations for this.
Antigenic determinants, normally camouflaged by
thymocytes, may have simply been exposed due to
the loss of lymphocytes; the stromal cells may be
responding directly to the effects of the irradiation;
the sudden loss of thymocytes may have caused an
upregulation in the stromal cell expression of these
antigens. In accordance with clearly established data
of previous studies (Takada et al., 1969; Huiskamp
et al., 1983; Huiskamp and van Ewijk, 1985;
Huiskamp et al., 1985), following irradiation there
was indeed a dramatic decrease in thymic mass and
collapse of the cortical stromal network through the
loss of immature thymocytes. The developmental
status of the remnant and regenerating T cells was
verified not only by the changes in CD3, CD4, and
CD8 profiles and the collapsed cortex, but also by
the loss of TSA-1/Sca-2 (MTS 35), ThB (MTS 33),
and HSA (MTS 37) positive cells. MTS 35 detects
the antigen TSA-1/Sca-2, which is expressed only
on immature thymocytes and is normally absent on
peripheral T cells (Godfrey et al., 1992). Similar to
HSA, ThB is present on most thymocytes, including
the mature (CD3/) CD4 and CD8 siffgle-positive
populations, but is absent on peripheral T cells
(Tucek et al., 1992). By days 3-4 PIrr, there was
virtually no staining for ThB and HSA. This was
surprising as phenotypically mature thymocytes
were still present. It is possible that these antigens
are radiosensitive or are cleaved by macrophage-
released proteases, but this would infer that the
stromal cells that are still stained by the mAbs
express different, more stable conformations of the
antigens. There is no evidence to suggest immigra-
tion of mature peripheral (HSA- and ThB- negative)
T cells into the irradiated thymus. The apparent
buildup of CD3/CD4-CD8 and CD3/CD4 CD8
thymocytes presumably indicates a lag between
phenotypic and functional (at least in terms of
migration ability) maturity or it reveals a population
of cells that may never leave the thymus reflecting
the very low migration rate (,,1%/day; Scollay et
al., 1980).
Given the dramatic loss of cortical thymocytes,
this could in principle expose the stromal antigens
and hence the appearance of increased expression.
This in part may be an explanation, but it does not
account for the selective nature of the upregulated
antigens and in any case would only be relevant in
the cortex, but several antigens were enhanced in
the medulla (MTS 9, 14, 16, 17, and 29). From a
purely technical viewpoint, it is also unlikely that
the lymphocytes would normally sterically mask
stromal antigens because they are nonoverlapping
independent cell types and examined in the same
plane of the section. Direct irradiation induced
enhancement of the stromal antigens is also unlikely
because we have observed similar, albeit less pro-
nounced, increases following thymocyte depletion
with hydrocortisone and 5-fluorouracil (unpub-
114 E.S. RANDLE-BARRETT and R.L. BOYD
lished observations). We thus favor the hypothesis
that although some of the increases may be part of
the "housekeeping" repair mechanisms following
architectural damage to the thymus, they are essen-
tially a direct response of the stromal-cell subsets to
specifically reestablish a microenvironment geared
to reinitiating or elevating basal levels of T-cell
differentiation. Indeed, such specific upregulation of
these molecules may be a generic feature of the
thymus undergoing elevated thymopoiesis because
we have found similar selective upregulation of
stromal antigens during early ontogenesis (E14) and
in the postcastration reversal of thymic atrophy
(Price et al., manuscript in preparation).
Within the first 2 days following irradiation, TN
thymocytes did not alter significantly, indicating
their relative resistance to the irradiation; as to
whether there were subtle shifts in the CD44/
CD25/c-kit defined TN subsets was not determined.
It is from within these TN cells that reconstitution
begins around day 4 is rapidly expanded by day 7
and is virtually complete by day 21. High levels of
[3H] TdR incorporation (Kadish and Basch, 1975;
Sharp and Thomas, 1975) and increased lympho-
cyte reactivity of the mAb MTS 35, 32, 37, and ThB
also indicated this to be a time of active cell division
and increased immature thymocyte content.
Based on the findings reported herein, a synopsis
of the intrathymic events following sublethal irra-
diation would be as follows. The initial and proba-
bly sole direct effect of the irradiation is the
immediate loss of immature thymocytes, excluding
the nondividing TN precursors. This causes an
associated collapse of the cortex, the epithelium of
which is still present, but as a compact zone of MTS
44 cells surrounding the medulla. From 24 to 72 h,
there was a dramatic increase in macrophages and/
or myeloid cells revealed by enhanced MAC-l, MTS
17, MTS 37, and MTS 28 (data not shown) staining,
presumably in response to the need to remove the
dead and dying thymocytes. This increase in phago-
cytic cells is in agreement with earlier reports (Du-
ijvestijn et al., 1982; Huiskamp et al., 1985). The
loss of thymocytes was also associated with a
marked increase in expression of antigens associated
with the vasculature. Because it is unlikely that
significant angiogenesis occurred during this time,
the apparent increase in vascular endothelium (MTS
12 was more likely due to it being exposed. There
was, however, an upregulation in the expression of
the secreted endothelial antigen detected by MTS 15
and the extracellular matrix associated with the
vasculature (MTS 16). Although the latter may be
part of a tissue rebuilding process, we have also
observed such endothelial activation in the regen-
erating male mouse thymus following castration
(Price et al., manuscript in preparation). Hence as
TN thymic precursors are localized around the
thymic endothelium, it is possible that the vascula-
ture initiates their proliferation and/or differentia-
tion, evident from day 4 PIrr. The dynamic nature of
the thymic stromal cells was further demonstrated
by the dramatic increase in MTS 9 expression,
which virtually stained the entire thymus from
24-72 h and gradually retracted with the increase in
newly generated T cells, to the predominantly med-
ullary epithelium pattern by day 6.
Hence, despite the widely held view that thymic
stromal cells are a sessile population, they are
capable of rapid alteration at both the cellular and
molecular levels in response to the sudden depletion
of thymocytes or need for renewed thymopoiesis.
The post-irradiation model provides a valuable
means of revealing this and we are currently using
it to test the functional significance of the antigens
by injecting the appropriate purified mAbs.
MATERIALS AND METHODS
Animals
CBA/CaH male mice 4-6 weeks of age were ob-
tained from the Monash University Central Animal
House. The mice were exposed to 7.5 Gy of whole
body y irradiation at a dose rate of 0.3 Gy/min, at
the Walter and Eliza Hall Institute using Eldorado 6
teletherapy unit (Atomic Energy of Canada, Com-
mercial Products) charged with 5000 Ci of 6Co.
Mice were subsequently killed at 12 h, days 1-14,
21, and 28 after the irradiation. Results are based on
a minimum of four independent experiments and a
pool of one to six mice per experiment, depending
on the time point.
Cell-Surface Staining
All labeling for CD3, CD4, and CD8 was performed
using anti-CD3-FITC (145-2Cll), anti-CD4 phyco-
erythrin (PE) (GK1.5), and anti-CD8-Biotin (B) (53-
6.7) (CD4 and CD8 both from Becton Dickinson,
CA). For three-color labeling, streptavidin-
phycoerythrin Texas red conjugate (TandemTM,
Southern Biotech, AL) was used to detect the
THYMIC RECONSTITUTION AFTER IRRADIATION 115
biotinylated mAb. To control for labeling efficiency,
freshly prepared thymocyte suspensions from nor-
mal adult mice were routinely used. For MTS mAb
thymocyte reactivity, cells were indirectly labeled
with the desired mAb followed by FITC-conjugated
sheep anti-rat Ig (Silenus Laboratories). Stained cells
were monitored using a FACScan (Becton Dickin-
son). Dead cells and nonlymphoid cells were ex-
cluded from data acquisition on the basis of 0 and
90 scatter profiles. Analyses were performed using
Lysys II research software (Becton Dickinson).
Immunohistology
Thymii were snap frozen on liquid nitrogen and 4
m sections were cut using a cryostat. MTS mAb
(Godfrey et al., 1990) labeling and the coexpression
of epithelial cell determinants were assessed by
double-labeling sections with the desired mAb and
a polyvalent rabbit anti-cytokeratin Ab (Dako,
Carpinteria, CA), respectively. Bound mAb was
revealed by FITC-conjugated sheep anti-rat Ig (Sile-
nus Laboratories) and TRITC-conjugated goat anti-
rabbit Ig (Silenus Laboratories). By using these
species combinations, no intermediate blocking
steps were necessary. For three-color immunofluo-
rescence, sections were labeled with anti-CD4-FITC,
anti-CD8-Biotin (both from Becton Dickinson, and
the polyvalent rabbit anti-cytokeratin Ab, washed
and stained with a mixture of streptavidin-
phycoerythrin Texas red conjugate (TandemTM,
Southern Biotech) and AMCA anti-rabbit Ig (Jack-
son Immunodiagnostics, WG). Stained sections were
washed and mounted under a coverslip using
veronal buffered glycerol (pH 8.6) and examined
using a Zeiss Axioskop fluorescence microscope. All
photography was performed using Kodak 1000
ASA print or 1600 slide films.
ACKNOWLEDGMENTS
The authors would like to thank Miss Anna Romeo and
Dr. Leon Martin for advice regarding FCM, Dr. Dale
Godfrey for his valuable discussion and photographic
contributions, and Dr. Carolyn Tucek-Szabo for her early
participation in this project. This work was supported by
grants from the Australian National Health and Medical
Research Council and the Anti-Cancer Council of Victoria.
(Received April 5, 1994)
(Accepted September 2, 1994)
REFERENCES
Adkins B., Gandour D., Strober S., and Weissman I. (1988). Total
lymphoid irradiation leads to transient depletion of the mouse
thymic medulla and persistent abnormalities among medullary
stromal cells. J. Immunol. 140: 3373-3379.
Anderson G., Jenkinson E.J., Moore N.C., and Owen J.J.T. (1993).
MHC class II-positive epithelium and mesenchyme cells are
both required for T-cell development in the thymus. Nature
362: 70-73.
Andrews P., and Boyd R.L. (1985). The murine thymic nurse cell:
An isolated thymic microenvironment. Eur. J. Immunol. 15:
36-42.
Boyd R.L., and Hugo P. (1991). Towards an intergrated view of
thymopoiesis. Immunol. Today 12: 71-79.
Boyd R.L., Tucek, CL., Godfrey D.I., Izon D.J., Wilson T.J.,
Davidson N.J., Bean A.G.D., Ladyman H.M., Ritter M.A., and
Hugo P. (1993). The thymic microenvironment. Immunol.
Today 14: 445-459.
Couture C., Amarante-Mendes G., and Potworowski E.F. (1992).
Tyrosine kinase activation in thymic epithelial tells: Necessity
of thymocyte contact through the gp23/45/90 adhesion com-
plex. Eur. J. Immunol. 22: 2579-2585.
Duijvestijn A.M., Kohler Y.G., and Hoefsmit E.C.M. (1982) In-
derdigitating cells and macrophages in the acute involuting rat
thymus. An electronmicroscopic study on phagocytic activity
and population development. Cell. Tissue Res. 224: 291-301.
Gao X., Nishimura T., Takeichi Y., Sudo T., and Habu S. (1993)
Thymic nurse cell clone supports the differentiation of
CD4-CD8- thymocytes into CD4/CD8 thymocytes in vitro.
Immunol. Lett. 35: 169-176.
Godfrey D.I., Izon D.J., Tucek C.L., Wilson T.J., and Boyd R.L.
(1990). The phenotypic heterogeneity of mousethymic stromal
cells. Immunol. 70: 66-74.
Godfrey D.I., Masciantonio M., Tucek C.L., Malin M.A., and Boyd
R.L. (1992). A novel thymocyte marker discriminating imma-
ture from mature thymocyte subsets. J. Immunol. 148: 2006-
2011.
Hiesch K.D., and Revesz L. (1979). Effect of cortisone and
x-irradiation on cellular depletion and regeneration in the
thymus of mice: Experimental discrimination between thymus
lymphocyte precursors in the bone marrow and in the thymus.
Path. Res. Pract. 164: 157-166.
Hugo P., Kappler J.W., Godfrey D.I., and Marrack P.C. (1992). A
cell line that can induce thymocyte positive selection. Nature
360: 679-682.
Huiskamp R., Davids J.A.G., and Vos O. (1983). Short- and
long-term effects of whole body irradiation with fission neu-
trons or X-rays on the thymus in CBA mice. Rad. Res. 95:
370-381.
Huiskamp R., and van Ewijk W. (1985). Repopulation of the
mouse thymus after sublethal fission neutron irradiation. I.
Sequential appearance of thymocyte subpopulations. J. Immu-
nol. 134: 2161-2169.
Huiskamp R., van Vliet E., and van Ewijk W. (1985) Repopulation
of the mouse thymus after sublethal fission neutron irradiation.
II. Sequential changes in the thymic microenvironment. J.
Immunol. 134: 2170-2178.
Kadish J.L., and Basch R.S. (1975). Thymic regeneration after
lethal irradiation: Evidence for an intra-thymic radioresistant T
cell precursor. J. Immunol. 114: 452-458.
Kanariou M., Huby R., Ladyman H., Colic M., Sivolapenko G.,
Lampert I., and Ritter M. (1989). Immunosuppression with
cyclosporin A alters the thymic microenvironment. Clin. Exp.
Immunol. 78: 262-270.
Kyewski B.A. (1986). Thymic nurse cells: Possible sites of T cell
selection. Immunol. Today 7: 374-379.
Kyewski B.A. (1991). Differential effects of anti-CD3 antibodies
116 E.S. RANDLE-BARRETT and R.L. BOYD
in vivo and in vitro on o and ,8 T cell differentiation. Curr.
Top. Microbiol. Immunol. 173: 65-69.
Molina T.J., Kishihara K., Siderovski D.P., van Ewijk W., Naren-
drsn A., Timms E., Wakeham A., Paige C.J., Hartmann K.U.,
Veillette A., Davidson D., and Mak T.W. (1992). Profound
block in thymocyte development in mice lacking p56LcK.
Nature 357:161-164.
Nagamine J., Takeda K., Tatsumi Y., Ogata M., Miyake K.,
Hamaoka T., and Fujiwara H. (1991). Role of a thymic stromal
cell clone in inducing the stage-specific differentiation of
various subpopulations of double negative thymocytes. J.
Immunol. 147: 1147-1152.
Nishimura T., Takeuchi Y., Ichimura Y., Gao X., Akatsuka A.,
Tamaoki N., Yagita H., Okumura K., and Habu S. (1990).
Thymic stromal cell clone with nursing activity supports the
growth and differentiation of murine CD4+8 thymocytes in
vitro. J. Immunol. 145: 4012-4017.
Palacios R., Studer S., Samaeidis J., and Pelkonen J. (1989).
Thymic epithelial cells induce in vitro differentiation of PRO-T
lymphocyte clones into tCRa,/T3+ and TCR,,8/T3+ cells.
EMBO J. 8: 4053-4063.
Palmer D.B., Viney J.L., Ritter M.A., Hatdat A.C., and Owen M.J.
(1993). Expression of the ofl T-cell receptor is necessary for the
generation of the thymic medulla. Dev. Immunol. 3: 175-179.
Philpott K.L., Viney J.L., Kay G., Rastan S., Gardiner E.M., Chae
S., Hayday A.C., and Owen M.J. (1992). Lymphoid develop-
ment in mice congenitally lacking T cell receptor cfl-expressing
cells. Science 256: 1448-1452.
Ritter M.A., and Boyd R.L. (1993). Development in the thymus: It
takes two to tango. Immunol. Today 14: 462-468.
Scollay R., Butcher E.C., and Weissman I.L. (1980). Thymus cell
migration. Quantative aspects of cellular traffic from the thy-
mus to the periphery in mice. Eur. J. Immunol. 10: 210-218.
Sharp J.G., and Thomas D.B. (1975). Thymic regeneration in
lethally X-irradiated mice. Radiat. Res. 64: 293-305.
Sharp J.G., and Thomas D.B. (1977). Origin of the radioresistant
precursor cells responsible for the initial phase of thymic
regeneration after X-irradiation. J. Retic. Soc. 22: 169-179.
Shores E.W., Van Ewijk W., and Singer A. (1991). Disorganization
and restoration of thymic medullary epithelial cells in T cell
receptor-negative scid mice: Evidence that receptor-bearing
lymphocytes influence maturation of the thymic microenviron-
ment. Eur. J. Immunol. 21: 1657-1661.
Shortman K., and Vremec D. (1991). Different subpopulations of
developing thymocytes are associated with adherent (Mac-
rophages) or nonadherent (Dendritic) thymic rosettes. Dev.
Immunol. 1: 225-235.
Surh C.D., Ernst B., and Sprent J. (1992). Growth of epithelial
cells in the thymic medulla is under the control of mature T
cells. J. Exp. Med. 176:611-616.
Takada A., Takada Y., Huang C.C., and Ambrus J.L. (1969).
Biphasic pattern of thymus regeneration after whole body
irradiation. J. Exp. Med. 129: 445-456.
Tatsumi Y., Kumanogoh A., Saito M., Mizushima Y., Kimura K.,
Suzuki S., Yagi H., Horiuchi A., Ogata M., Hamaoka T., and
Fujiwara H. (1990). Differentiation of thymocytes from
CD3-4-8- through CD3-4-8 into more mature stages induced
by a thymic stromal cell clone. Proc. Natl. Acad. Sci. USA 87:
2750-2754.
Tucek C.L., Godfrey D.I., and Boyd R.L. (1992). Five novel
antigens illustrate shared antigenicity between mouse thymic
stromal cells, thymocytes and peripheral lymphocytes. Int.
Immunol. 4: 1021-1030.
Vacari A., Abehsira-Amar O., Papiernik M., Boyd R.L., and Tucek
C.L. (1994). MTS 32-monodonal antibody defines CD4/8
thymocyte subsets that differ in their maturation level, lym-
phokine secretion, and selection patterns. J. Immunol. 152:
333-335.
Van Ewijk W. (1988). Cell surface topography of thymic microen-
vironments. Lab Invest. 59: 579-590.
Van Ewijk W., Shores E.W., and Singer A. (1994). Crosstalk in the
mouse thymus. Immunol. Today 15:214-217.
Watanabe Y., Mazda O., Aiba Y.K.I., Gyotoku J., Ideyama S.,
Miyazaki J., and Katsura Y. (1992). A murine thymic stromal
cell line which may support the differentiation of CD4-8-
thymocytes into CD4+8-c,8 T cell receptor positive T cells. Cell
Immunol. 142: 385-397.

				

